# Vats lobectomy for lung cancer. What has been the evolution over the time?

**DOI:** 10.3389/fonc.2023.1268362

**Published:** 2024-01-08

**Authors:** Emanuela Re Cecconi, Giuseppe Mangiameli, Matilde De Simone, Ugo Cioffi, Giuseppe Marulli, Alberto Testori

**Affiliations:** ^1^ Division of Thoracic Surgery, Department of Thoracic Surgery, Tor Vergata University Polyclinic, Rome, Italy; ^2^ Division of Thoracic Surgery, IRCCS Humanitas Research Hospital, Milan, Italy; ^3^ Department of Biomedical Sciences, Humanitas University, Milan, Italy; ^4^ Department of Surgery, University of Milan, Milan, Italy

**Keywords:** uniportal VATS, video-assisted thoracic surgery, lung cancer, VATS (video-assisted thoracic surgery), thoracic surgery

## Abstract

Video assisted thoracic surgery (VATS) lobectomy is the treatment of choice for early-stage lung cancer. It is safe and effective compared to open surgery, as demonstrated by a large body of scientific evidence over the last few decades. VATS lobectomy’s evolution was driven by the need to decrease post-operative pain by reducing the extent of surgical accesses, maintaining the same oncological efficacy of open lobectomy with less invasiveness. VATS lobectomy just turned 30 years old, evolving and changing significantly from its origins. The aim of this mini review is to retrace the history, starting from a multiport approach to a single port approach. At the end of this mini review, we will discuss the advanced and the future challenges of the technique that has revolutionized thoracic surgery.

## Introduction

Video assisted thoracic surgery (VATS) lobectomy is defined as video-guided anatomical resection with individual ligation of the lobar vessels and bronchus. VATS is defined as a non-rib spreading thoracic procedure, characterized by complete thoracoscopic visualization as opposed to the direct visualization of open procedures (1). At the end of 20th Century, the minimally invasive approach by VATS for major lung resections (i.e., lobectomy) started a revolution in thoracic surgery, nowadays routinely performed by surgical teams all over the world.

Dr. Hans Christian Jacobeus was the first physician who performed a thoracoscopy. In 1910 he introduced a light beam in a cystoscope to explore the pleural cavity. He wanted to lyse pleural adhesions in patients with tuberculosis, a common condition at the time, to allow re-expansion of affected lungs (2). From that moment onward, thoracoscopy was used for both diagnostic and therapeutic procedures of pleural diseases (3). In the early ‘90s the improvements in biomedical sciences and the advent of mechanical staplers, electrosurgical and endoscopic instruments made VATS major resections a revolutionary real possibility. Overtime, the development of VATS lobectomy was based on two milestones, which are the standardization of the technique by reducing invasiveness and the demonstration of oncological effectiveness.

The first structured studies performed by Kirby, Lewis and Roviaro date back to the beginning of the 1990s and since then the technique spread and developed significantly. Regarding the technical evolution, since the first VATS lobectomies were performed with two small incisions and one 6-8 cm mini-thoracotomic access (4), subsequently a biportal (5) and then uniportal (6) VATS lobectomy developed, with the latest subxiphoid approach proposal (7).

At the same time, several prospective and retrospective studies (8, 9) demonstrated that VATS lobectomy is safe and oncologically comparable to the standard open one, reporting similar mortality and overall survival rates (10). Additionally, VATS lobectomy is associated with shorter length of stay, less pain and lower morbidity rate when compared with thoracotomy (11). In its 30 years of history VATS lobectomy revolutionized lung- cancer surgery and treatment, offering a safe, less painful and equally efficient alternative to open surgery. Because of the minor trauma created by endoscopic surgery, VATS lobectomy enlarged the number of patients fit for surgery.

Thus, in the light of these outstanding evidences, VATS lobectomy gained the ‘grade 2C’ recommendation as a preferred technique over open surgery for the treatment of the early-stage NSCLC by the American College of Chest Physicians evidence-based guidelines in 2013 (12).

In this review we present the technical evolution of VATS lobectomy, from a multi-port approach to a single-port approach, exploring the challenges that surgeons successfully overcame also thanks to technological advancements.

## Triportal vats lobectomy

Northern Europe, with the Copenhagen and the Edinburgh experiences, was the land of the anterior and posterior three-port approaches.

**Figure 1:**
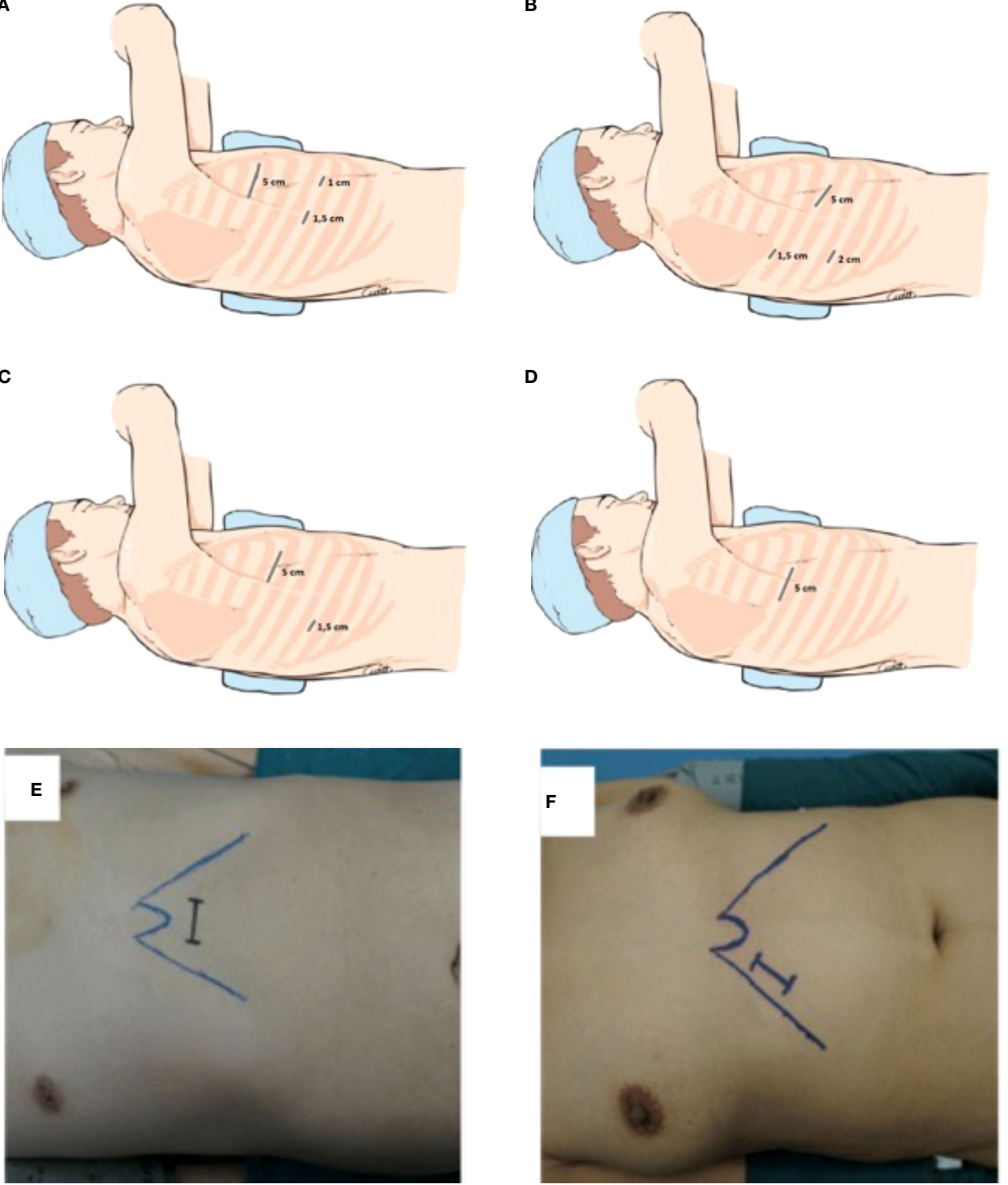
**(A)** Triportal anterior approach, **(B)** Triportal posterior approach, **(C)** Biportal approach, **(D)** Uniportal approach, **(E)** Subxiphoid approach, **(F)** subcostal approach.

Copenhagen anterior approach gets its name from the position of the incisions. It was described in 2012 by Hansen et al. (13) who performed more than 1000 VATS lobectomies at the Rigshospitalet, Copenhagen University Hospital, starting from the early 2000s. It provides the use of an utility incision located anterior to the latissimus dorsi muscle and two lower incisions. In particular, the surgeon and the assistant are placed anteriorly to the patient, with the surgeon cranially. The scrub nurse is opposite to the assistant. At first, a 5-cm utility access is performed between the lower angle of the scapula and the breast, at the level of 4th or 5th intercostal space. Lower, a 1-cm camera port is positioned just above the diaphragm, anteriorly to the hilum, and a 1,5 cm access is positioned more posteriorly (**Figure 1A**). The camera is usually introduced through the anterior access. Both the utility and the posterior access are used for instrument manipulation and a mix of endoscopic and standard open instrumentation is used. The preferred camera is 10 mm30°. The dissection is performed starting from the hilum anteriorly.

The anterior access allows a good triangular view of the pulmonary hilum. During this procedure, the structures are usually divided from anterior to posterior. The advantages of this standardized anterior approach are a good visualization of the hilum and an intuitive anatomical angulation, resulting in easy manipulation of the hilum and the great vessels which are the first structures to be transected (13). Furthermore, the utility incision is directly over the hilum and the pulmonary vessels, allowing to easily clamp the major vessels in case of the major bleeding; the surgeon doesn’t need to change its position or the site of incision if a conversion is required. Last but not least the reproducibility of this technique is the main advantage advocated by the Danish surgical group.

The Edinburgh posterior approach was described by Richard and Colleagues in 2012 (14). This approach enables a good visualization of the posterior hilum. The idea of a posterior approach developed from the experience of postero-lateral thoracotomy and mimics the steps of posterior open lobectomies. The surgeon and the assistant are positioned posteriorly to the patient who is in lateral decubitus. The scrub nurse is on the opposite side. The 5-cm utility port is positioned at the level of the 6th or 7th intercostal space anterior to the latissimus dorsi muscle, instead of the 4^th^ or 5 th intercostal spaces. Then, a 1,5 cm camera port is positioned in the auscultatory triangle and an additional 2 cm port is located where the midaxillary line meets the upper third of the anterior utility port (**Figure 1B**). In this approach the adopted thoracoscopy is 0o rather than 30o.The posterior approach allows good visualization of the posterior hilum, enabling direct control of the pulmonary artery, bronchial branches, a better visualization of lymph nodes, and a safer dissection due to the tips of the instruments coming towards the camera (14). Compared to the anterior approach, the main technical difference is the order of dissection which usually is performed from the posterior to anterior, by opening up the fissure to identify and isolate pulmonary arterial branches. A mix of endoscopic and standard open instruments is employed. Arterial vessels are dissected and isolated through the oblique fissure using long artery forceps, curved forceps and endo-dissectors. As in the techniques previously described major vessels, bronchi and lung parenchima are sectioned using dedicated stapling devices to ensure hemostasis and aerostasis.

**Table 1** resumes the most important studies evaluating surgical results of triportal approach reported in last ten years.

**Table 1:** Studies with more than 100 cases, describing short term results for each VATS modality.

Authors	Approach	Years	N° of patients	Complications (n)	Conversion	Length of stay (median)	30 days Mortality (n)
Onaitis (15)	Biportal	2006	500	119	8 (1,6%)	3	6 (1,2%)
Kim (16)	Triportal posterior	2010	740	76	36 (4,9%)	NA	9 (1,3%)
Hansen (13)	Triportal anterior	2010	169	103	13 (7,7%)	6	0 (0%)
Gonzales Rivas (17)	Uniportal	2013	102 (5 pneumonectomies)	14	5 (4,9%) (3 to open, 2 to biportal VATS)	3	0 (0%)
Song (18)	subxhypoid	2016	105	11	8 (2 to open, 6 to standard VATS	5,4	0%

In the table we reported 5 case series, one for each technique, with more than 100 cases. (13, 15, 17, 18). Most studies are the initial results of the authors or centers standardizing the technique.

## Bi portal vats lobectomy

Thomas D’Amico and Colleagues from Duke University first described in 2004 and then standardized the two-ports approach for VATS lobectomy (5, 15).

According to his description, a 1-cm thoracoscope port is placed in the 7th or 8th intercostal space in the mid-axillary line. A second incision, 4,5-6 cm in length, is placed anteriorly, below the inferior margin of the pectoralis muscle at 4th or 5th intercostal space (**Figure 1C**) and it is used for dissection and specimen retrieval. D’Amico explains that the location of the incision allows a good visualization of the hilum, avoiding competition between instruments. Moreover, both ports allow for insertion of stapling device, favoring for the best angulations (5, 15).

Patient is positioned in lateral decubitus, the operator and the assistant are placed anteriorly, while the scrub nurse is on the opposite side. The camera is placed in the lower access. The access warrants a good direct visualization on the anterior hilum, while to dissect the hilum posteriorly it is necessary to retract the lung. As for the triportal approach, dedicated endoscopic instrumentation is required.

Nowadays the bi-portal approach has been largely adopted in many Centers, it is accepted as a valid alternative to open surgery also for complex interventions such as pneumonectomy, sleeve lobectomy, and anatomic lung resections after induction treatment in stage IIIA NSCLC. Surgical results are reported in **Table 1**. Biportal VATS is considered by some “a bridge toward uniportal VATS” (20).

## Uniportal vats lobectomy

In 2004, when D’Amico described the two-port VATS lobectomy technique, Rocco and colleagues published the first single-port attempts by adopting an uniportal approach (21). They reported 109 cases of uniportal VATS, mostly pleural biopsies and wedge resections and no major lung resections. Yet, they proposed the uniportal approach as a feasible way to reduce post-operative pain compared with the multiportal VATS.

In 2010 Diego Gonzales Rivas from Coruna University Hospital performed the first uniportal VATS lobectomy and in 2012 published the initial results of his experience with the uniportal approach. At the beginning, he also pointed out that the uniportal approach was easier and more feasible for the lower lobes, but when performed by a skilled or trained surgeon, it is adequate for all anatomic lung resections (6, 20).

In 2013 Luca Bertolaccini, an Italian physician with a background in physics, published an interesting paper reporting the geometrical characteristics of uniportal VATS. He concluded that the angle of standard three port VATS interferes with the optical source and creates an unfavorable torsion angle. On the contrary, uniportal VATS approaches the lesion in a sagittal way, preserving the depth of intraoperative visualization. Instruments create a plane that is more similar to open visualization (22).

Uniportal VATS lobectomy is performed using a single 3 to 5 cm incision at the fifth intercostal space on the anterior axillary line (**Figure 1D**). The modern thoracic surgical team includes one surgeon who operates together with the first assistant who holds the camera and a scrub nurse. The surgeon and his assistant should be positioned in front of the patient in order to have the same thoracoscopic vision during all steps of the procedure for more coordinated movements. The scrub nurse is located on the opposite side of the operating table. Single port VATS lobectomy can be performed with conventional instruments, but the use of especially adapted instrumentation with distal articulation, articulated staplers, vascular clips, modern energy devices and high definition 30° cameras can facilitate the surgeon in performing a successful uniportal VATS lobectomy. The camera is placed at the anterior end of the incision during vessels and bronchus dissection and is moved to the posterior end for lymphadenectomy (6). An experiences anaesthetist should also be present in case of complications. Correct retraction of the lung parenchyma and bimanual instrumentation are key points (23). The two main advantages of uniportal VATS approach are a reduction of the post-operative pain due to the strain on a single intercostal space/nerve and a vision angle similar to open thoracotomy. Uniportal VATS lobectomy is a trend that is becoming increasingly popular, and several authors are publishing results reporting the successful outcome also in complex resections (24, 25). Moreover, increasing amount of data is showing the safety and oncological effectiveness (26, 27). **Table 2** reports comparative studies between uniportal and multiportal approach in the last 10 years.

**Table 2:** Comparative studies with more than 100 cases between uniportal and multiportal vats lobectomies in the last 10 years.

Authors	Approch	Years	N° of patients	Complications	Conversion	Lenght of stay (median)	30 days Mortality (n)
Shen (27)	Uniportal	2013-2014	100	4 (4%) *	1	4,7	0
	Triportal	2013-2014	100	7 (7%) *	2	5,3	0
Tosi (28)	Uniportal	2014-2017	172	35 (20%)	NA	4	0
	Triportal	2014-2017	1808	373 (20%)	NA	5	0
Al-Ameri (29)	Uniportal	2016	122	7 (6%)	12 (9%)	3,5	0
	Multiportal	2018	211	13 (6%)	0	3,5	1

Three comparative studies are reported, comparing uniportal vs multiportal vats. All the studies include more than 100 cases for each technique.

* non including post operative arrhythmias (27–29).

## Purely thoracoscopic lobectomy

This mini-invasive approach was first described by Dominique Gossot in 2009. He presented a cases series with 69 patients who underwent totally endoscopic major pulmonary resections between 2007 and 2009 (30).

This approach employs 2-4 thoracoports and no utility access. To retrieve the surgical specimen a 3-4 cm access could be located anywhere in the hemithorax at the end of intervention, the Authors argued that this access is less traumatic given the smaller dimensions and the short usage time. A camera holder may also be used to minimize instrument conflicts, of instruments as well as dedicated fully endoscopic instruments (31). During the procedure, a CO2 insufflation favoring further collapse of the lung, provides a larger working area.

## Subxyphoideal uniportal vats lobectomy

Starting from a traditional intercostal uniportal approach, Liu and colleagues from the Taipei University Hospital presented the first case of thoracoscopic left lower lobectomy in 2014 by using a 4 cm subxiphoid incision lifting the sternocostal margin with a retractor to expose the area (32). The reported advantages of this approach were the ability to use different size of instruments with wide freedom of movement as there is no limitation by the ribs and avoiding the post-operative pain typically experienced due to bruising of the intercostal nerves (32).

Diego Gonzales Rivas described a variation of this approach that provided the xyphoid process resection (**Figure 1E**). The dissection is carried out by using a long energy device, dedicated instruments and longer articulated mechanical staplers. He concluded that this approach is technically more challenging than other classical thoracoscopic approach thus good skills with traditional endoscopic approaches is essential (7). The main advantages of subxyphoid vs intercostal uniportal approach are the decreased post-operative pain and the possibility to perform bilateral surgeries. On the other hand, an increased risk of bleeding has been reported in subxyphoid VATS lobectomy. As mentioned, the subxyphoid approach requires highly experienced thoracoscopic surgeons and adequate instrumentation (7).

### Subcostal vats lobectomy

The Subcostal access proposed by Al Sawalhi et al. is an alternative to subxiphoid one (33). The access is a 4-5 cm uniport, parallel to the costal arch in which the rectus abdominis is dissected along the subcostal arch (**Figure 1F**). As for the subxiphoid approach, longer instrumentation is the key for good manipulation and dissection of the lung parenchyma and vessels. Convincing and solid literature on the topic is still lacking. The advantages are comparable with those of the subxiphoid approach, with the decrease in post operative pain as the main goal.

### Awake vats surgery

Alongside the recent surgical and technological advances that have provided dedicated instruments and allowed smaller and fewer cutaneous incisions, some attempts at simplifying the anaesthetic procedures and decreasing their adverse effects have been made. Traditionally intubated general anesthesia with single-lung mechanical ventilation is considered the standard approach for thoracic surgery, it allows the surgeon to isolate the operative lung, protect the main airway and grant optimal surgical conditions. However intubated general anesthesia is related to several complications such as airway trauma, ventilation-induced lung injury, impaired cardiac performance, nausea and vomiting and carries a higher risk for patients considered unfit because of old age or significant comorbidities (34). In this scenario non-intubated surgery, an approach already adopted before the introduction of selective lung ventilation, has been considered a valid alternative (35).

A recent survey suggested that it is still performed for simple thoracoscopic procedures, such as pleural, mediastinal and lung biopsies (36). These promising results encouraged surgeons and anaesthesiologists to use non-intubated thoracic surgical techniques for the more challenging and technically demanding anatomical resections, such as lobectomies. The challenge of performing a lobectomy when the patient is awake lies in the need to dissect, isolate and suture delicate structures in conditions of discomfort such as the lack of stillness of the surgical field and the relentless inflation and deflation of the lung. Consequently, such interventions require experienced and well-trained VATS surgeons along with competent anaesthesiologists. More recently a metanalysis aimed at assessing safety, feasibility and oncological outcomes of non-intubated thoracoscopic lobectomies for NSCLC. Interestingly, despite the limitation of only 3 papers included, awake and intubated thoracoscopic lobectomies for resectable NSCLC seem to have comparable perioperative and postoperative outcomes. Nevertheless, the oncological implications of the non-intubated approach should be considered. The long-term benefits for patients with lung cancer need to be carefully assessed because operative skills are undeniably essential and might vary reasonably among surgeons (37).

## Discussion

VATS lobectomies have recently turned 30 years-old. During this lifespan, technical and technological evolutions have been continuous, increasing the safety and the accuracy of the procedures. At the beginning of this story, considering the open thoracotomic approach as the benchmark, the main concerns regarding the VATS approach for lobectomy raised from the uncertainties on oncological efficacy and the accuracy and extension of lymph node dissection. Literature on this topic is various with some authors suggesting evidences of an increased nodal upstaging in open vs VATS lobectomy (38), while others authors did not notice significant differences in nodal upstaging when comparing the two techniques (38). The oncological value of these findings, however, has not been demonstrated, yet considering that no prospective randomized trials evaluating VATS surgery relative efficacy and oncologic equivalence in comparison to open surgery are yet available. The recently published prospective randomized trial named VIOLET answered some of these questions reporting on a total of 503 patients randomized, 247 of which underwent VATS lobectomy and the remaining 256 traditional open lobectomy (11). The first group was associated with better physical functioning at 5 weeks (primary outcome), less post-operative pain, reduced risk of adverse events, and no difference in cancer free survival at 52 weeks. This trial represents an important milestone, being the first large randomized trial on the topic.

As we have exposed in this article, VATS lobectomy is an evolving technique and the differences between the approaches are fundamental.

Several studies have shown as the triple port access is safe, oncologically equal to muscle sparing open thoracotomy, less painful for the patient and with better aesthetic results (39). Moreover, it offers a wide range of angulations when compared to single or double port VATS.

Recently, moving from the effort to further reduce invasiveness other mini-invasive approaches have been experimented, thus bi-portal and single portal approaches are increasingly replacing the traditional triportal VATS. These new approaches arise from the idea that decreasing the number of accesses and consequently reducing the number of damaged intercostal nerves, an additional advantage would be obtained in term of postoperative pain. Both bi-portal and single portal approach share, other than the reduction of incisions, the main advantage to maintain the same anterior approach of the open surgery and the possibility to palpate the lung.

Similarly, purely thoracoscopic lobectomies with a mini-thoracotomy only for extraction of the lobe has been proposed by several authors to overcome the Achilles heel of utility mini-thoracotomy which represents the common denominator of all the techniques described so far. On the other side, the main limitations of this approach are the longer operative time when compared to open thoracotomy or standard VATS technique and a very long learning curve which may justify the fact that this approach has not significantly spread. Comparably, the subxiphoid uniportal approach, by avoiding an intercostal incision, have as main advantage to decreased post-operative pain as well as allowing the possibility to perform bilateral surgeries but high experienced thoracoscopic surgeons and an adequate instrumentation are indispensable. Subxiphoid VATS still needs to be standardized, and some issues regarding safety are to be clarified. Moreover, the learning curve may be significantly longer with respect to intercostal uniportal VATS.

Biportal VATS is still a very common technique taught in universities worldwide assuring several advantages such as: less postoperative pain, shorter chest tube duration and subsequent length of stay, fewer overall complications, better compliance with adjuvant chemotherapy, faster return to full activity, and greater preservation of pulmonary function (40).

Nowadays, uniportal VATS lobectomy seems to be a winning technique, combining lower post-operative pain, good visualization of the pulmonary hilum, safety and efficacy. Studies focused on uniportal VATS learning curve for lobectomy are encouraging showing as it is without unacceptable complication rates and has a declining surgery duration over time for thoracic surgeons with experience in multiportal video-assisted thoracoscopic lobectomies. However, it remains unknown when the different stages of mastery are completed (41). A recent study comparing uniportal and multiportal non-intubated thoracoscopic anatomical resection for non-small cell lung cancer (NSCLC) showed as oncological outcomes such as recurrence-free and overall survival remained uncompromised (42).

Today, an increasing number of studies have reported the adoption of vats even in more complex cases such as tumor greater than 5 cm (43) or for advanced stage III NSCLC (44) showing as this mini-invasive approach remains feasible and effective for curative lobectomy for NSCLC but further validations from well-designed prospective studies are required. Furthermore, a new challenge will be represented by the increasing number of patients affected by resectable NSCLC after neoadjuvant immunotherapy and/or target therapy with initial studies showing that VATS lobectomy was not associated with an increased likelihood of the need for thoracotomy, conversion to open lobectomy, or inferior perioperative outcomes (45).

However, despite the undoubted advantages, VATS lobectomy has not been adopted widely. For example, it is currently estimated that VATS lobectomy rate is 30–40% in the USA, 30% in Europe, 50% in Italy, 65% in Denmark, and 29% in Great Britain and Ireland (46).

Probably the main explication to this limited widespread was the introduction of the Robotic Assisted Thoracic Surgery (RATS) over the past twenty years with the early experience with da Vinci robot (Intuitive Surgical, Sunnyvale, CA, USA) which showed that this minimally invasive approach is feasible and safe (19). The results of RATS are comparable to VATS but, at the same time, provide several typical and specific robotic advantages compared to VATS such as: binocular visualization allowing an excellent high-definition, three-dimensional view of the operating field, the degrees of freedom of robotics instruments that overcome several technical limitations of VATS due to the poor maneuverability of the straight rigid instruments through the rigid chest wall (47).

At the state of the art, there are no randomized trials comparing the two techniques and most comparative studies do not distinguish between different VATS techniques (48, 49). Most of the current data is based on case series and comparisons to historical cohorts or databases.

However, it is probably important to mention the results of a recent prospective international randomized control trial comparing the perioperative outcome and surgical radicality of the robotic approach with those of traditional video-assisted surgery in the treatment of the early-stage NSCLC. The results of this trial demonstrated that RATS was not superior to VATS considering the perioperative outcome for an early-stage NSCLC (50).

Indeed, comparative studies between VATS, RATS and open surgery are lacking, with most of them investigating short-term outcomes and providing poor evidence of comparable long-term oncological results. However, a recent paper of Casiraghi et al. reported no differences in overall survival and cancer-specific survival between VATS, RATS and open lobectomy for stage I NSCLC patients; even if in VATS, the incidence of recurrences, in particular local recurrences, was higher than in RATS and in open surgery (51). Anyway, several limits remain typical of RATS if compared to VATS approach, such as the high costs, the availability in peripherical structures and longer learning curve in surgeons without solid mini-invasive background (52).

## Conclusion

Since introduction of VATS lobectomy both technological advancement and refinement of surgical instruments have allowed a progressive evolution until confirmed as a preferred technique over open surgery for the treatment of the early-stage NSCLC. Its widespread is favored by the current era of lung cancer screening diagnosing a large proportion of early lung cancer cases ideal for VATS approach. In several thoracic surgery departments, VATS lobectomy has become the dominant approach with most centers using an anterior utility incision with one or two adjunctive ports. However, VATS lobectomy has not been adopted widely, probably due to the recent introduction of Robotic Assisted Thoracic Surgery (RATS) which seems comparable to VATS in term of feasibility and safety although burdened by higher costs, lower availability and longer learning curve in not experienced mini-invasive thoracic surgeons. Only the coming years will tell us what the direction of the minimally invasive lobectomy will be.

## Author contributions

AT: Validation, Visualization. EC: Writing – original draft. GMan: Writing – original draft, Writing – review & editing. MD: Supervision, Validation. UC: Supervision, Validation. GMar: Writing – review & editing.
